# Epidemiology of carbapenem-resistant and extended-spectrum beta-lactamase-producing Enterobacterales in US children, 2016–2020

**DOI:** 10.1017/ash.2023.231

**Published:** 2023-09-29

**Authors:** Heather Grome, Julian Grass, Nadezhda Duffy, Sandra Bulens, Jesse Jacob, Gillian Smith, Lucy Wilson, Elisabeth Vaeth, Bailey Evenson, Ghinwa Dumyati, Rebecca Tsay, Erin C. Phipps, Kristina Flores, Christopher Wilson, Christopher Czaja, Helen Johnston, Ruth Lynfield, Sean O’Malley, Meghan Maloney, Nicole Stabach, Joelle Nadle, Alice Guh

## Abstract

**Background:** The Centers for Disease Control and Prevention’s Emerging Infections Program conducts active laboratory- and population-based surveillance for carbapenem-resistant Enterobacterales (CRE) and extended spectrum beta-lactamase-producing Enterobacterales (ESBL-E). To better understand the U.S. epidemiology of these organisms among children, we determined the incidence of pediatric CRE and ESBL-E cases and described their clinical characteristics. **Methods:** Surveillance was conducted among children <18 years of age for CRE from 2016–2020 in 10 sites, and for ESBL-E from 2019–2020 in 6 sites. Among catchment-area residents, an incident CRE case was defined as the first isolation of *Escherichia coli*, *Enterobacter cloacae* complex, *Klebsiella aerogenes*, *K. oxytoca*, or *K. pneumoniae* in a 30-day period resistant to ≥1 carbapenem from a normally sterile site or urine. An incident ESBL-E case was defined as the first isolation of *E. coli, K. pneumoniae*, or *K. oxytoca* in a 30-day period resistant to any third-generation cephalosporin and non-resistant to all carbapenems from a normally sterile site or urine. Case records were reviewed. **Results:** Among 159 CRE cases, 131 (82.9%) were isolated from urine and 19 (12.0%) from blood; median age was 5 years (IQR 1–10) and 94 (59.1%) were female. Combined CRE incidence rate per 100,000 population by year ranged from 0.47 to 0.87. Among 207 ESBL-E cases, 160 (94.7%) were isolated from urine and 6 (3.6%) from blood; median age was 6 years (IQR 2–15) and 165 (79.7%) were female. Annual ESBL incidence rate per 100,000 population was 26.5 in 2019 and 19.63 in 2020. Incidence rates of CRE and ESBL-E were >2-fold higher in infants (children <1 year) than other age groups. Among those with data available, CRE cases were more likely than ESBL-E cases to have underlying conditions (99/158 [62.7%] versus 59/169 [34.9%], P<0.0001), prior healthcare exposures (74/158 [46.8%] versus 38/169 [22.5%], P<0.0001), and be hospitalized for any reason around time of their culture collection (75/158 [47.5%] versus 38/169 [22.5%], P<0.0001); median duration of admission was 18 days [IQR 3–103] for CRE versus 10 days [IQR 4–43] for ESBL-E. Urinary tract infection was the most frequent infection for CRE (89/158 [56.3%]) and ESBL-E (125/169 [74.0%]) cases. **Conclusion:** CRE infections occurred less frequently than ESBL-infections in U.S. children but were more often associated with healthcare risk factors and hospitalization. Infants had highest incidence of CRE and ESBL-E. Continued surveillance, infection prevention and control efforts, and antibiotic stewardship outside and within pediatric care are needed

**Disclosure:** None

